# Marital status and cause-specific mortality: A population-based prospective cohort study in southern Sweden

**DOI:** 10.1016/j.pmedr.2023.102542

**Published:** 2023-12-09

**Authors:** Martin Lindström, Mirnabi Pirouzifard, Maria Rosvall, Maria Fridh

**Affiliations:** a Social Medicine and Health Policy, Department of Clinical Sciences in Malmö and Centre for Primary Health Care Research, Lund University, S-205 02 Malmö, Sweden; b Department of Community Medicine and Public Health, Sahlgrenska Academy, Institute of Medicine, University of Gothenburg, Sweden

**Keywords:** Marital status, Mortality, Cardiovascular mortality, Cancer mortality, Health-related behaviors, Generalized trust in other people, Sweden

## Abstract

The aim was to investigate associations between marital status and mortality with a prospective cohort study design. A public health survey including adults aged 18–80 was conducted with a postal questionnaire in southern Sweden in 2008 (54.1% participation). The survey formed a baseline that was linked to 8.3-year follow-up all-cause, cardiovascular (CVD), cancer and other cause mortality. The present investigation entails 14,750 participants aged 45–80. Associations between marital status and mortality were investigated with multiple Cox-regression analyses. A 72.8% prevalence of respondents were married/cohabitating, 9.1% never married, 12.2% divorced and 5.9% widows/widowers. Marital status was associated with age, sex, socioeconomic status (SES) by occupation, country of birth, chronic disease, Body Mass Index (BMI), health-related behaviors and generalized trust covariates. Never married/single, divorced, and widowed men had significantly higher hazard rate ratios (HRRs) of all-cause mortality than the reference category married/cohabitating men throughout the multiple analyses. For men, CVD and other cause mortality showed similar significant results, but not cancer. No significant associations were displayed for women in the multiple analyses. Associations between marital status and mortality are stronger among men than women. Associations between marital status and cancer mortality are not statistically significant with low effect measures throughout the multiple analyses among both men and women.

## Highlights

• Being married/cohabitating is associated with better health globally.
• This population-based study investigated cause-specific mortality collapsing married and cohabitating.
• Married/cohabitating had lower all-cause, CVD and other mortality among men than women.
• Associations between marital status and cancer were not significant for both sexes.
• Married/cohabitating and widowed have higher prevalence of trust than unmarried/single and divorced.

## 1 Introduction

**Fig. 1:**
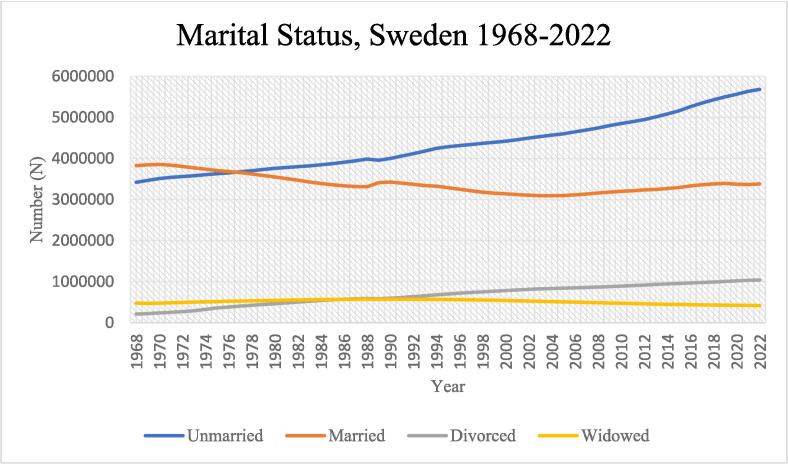
Swedish adult population by marital status 1968–2022. Source: Statistics Sweden (SCB), Population by region, marital status, age and sex. Year 1968–––2022. PxWeb (scb.se).

Marital status is associated with health around the world. Marriage/cohabitation is associated with better health and lower mortality (Liu et al., 2018, Chen et al., 2020, Leung et al., 2022). The unmarried have higher mortality rates than the married, even after adjustments for a variety of socioeconomic characteristics (Metsa-Simola and Martikainen, 2013, Manzoli et al., 2007). All-cause mortality rate, cardiovascular disease (CVD) mortality rate, CVD incidence rate and CVD risk factor prevalence are higher among the unmarried (Wong et al., 2018). Never married, divorced and widowed groups have higher mortality rates than married/cohabitating, but these associations are stronger among men who seem to benefit more than women from being married (Wang et al., 2020, Robards et al., 2012, Manzoli et al., 2007). Still, some studies have shown a lower risk of ischemic heart disease (IHD) mortality rate among married women compared to not married women (Floud et al., 2014). In a previous study in Scania with a 5.3-year follow-up, these associations between marital status and all-cause mortality rates were statistically significant among men but not among women, showing significantly higher hazard rate ratios for never married/single, divorced and widowed men compared to married/cohabitating men (Lindström and Rosvall, 2019), although cause-specific mortality was not investigated. In Sweden, age of marriage has increased from just below 25 years in the late 1960s and early 1970s to presently 35 years. Divorce rates increased dramatically in the 1980 s but have since then remained comparatively stable. Age at second marriage, if applicable, was approximately 40 years around 1970 and is currently 48 years (Statistics Sweden (SCB). Medelålder vid giftermål efter kön. År, 2017, Statistics Sweden (SCB). Befolkningsstatistik i sammandrag, 2022; see also Fig. 1). Same sex couples were granted the right to registered partnership in Sweden in 1995. Fourteen years later, in 2009, marriage was granted to same sex couples. Still, same sex couples are a numerically small group (Lindström and Rosvall, 2020).

Although associations between marital status and mortality are well known, several important issues remain. Most earlier studies displayed severe problems separating cohabitating from unmarried groups living alone (Ben-Schlomo et al., 1993, Berntsen and Kravdal, 2012). Given current patterns of cohabitation without marriage in Sweden, it is important to separate the unmarried cohabitating from unmarried living alone. Second, while the associations between marital status and all-cause mortality, CVD mortality and CVD risk factors are well-documented (Wong et al., 2018), the associations with cancer mortality have been found to be weaker, not statistically significant and have overall been less investigated (Wang et al., 2020).

Marriage has been suggested to be beneficial for health by the pathways of selection in younger ages, i.e. entry into marriage is facilitated by higher socioeconomic status in terms of education, income and occupation (Rendall et al., 2011), selection out of marriage for reasons of poor health and risky behaviors (Umberson, 1987), and protection (Waldron et al., 1996). The health benefits of protection would be particularly beneficial for spouses outside the workforce (Waldron et al., 1996). Marriage enhances health-promoting health-related behaviors and health beliefs (Franke and Kulu, 2018).

Marital status has in previous studies based on the public health surveys in Scania been demonstrated to be associated with higher prevalence of daily smoking (Lindström, 2010), health locus of control which influences health-related behaviors (Lindström and Rosvall, 2012a, Lindström and Rosvall, 2012), general and mental health (Lindström, 2009; Lindström and Rosvall, 2012b), and generalized trust in other people (Lindström, 2012). All these studies displayed significantly higher effect estimates of daily smoking, external health locus of control (among never married/single men), poor health and low generalized trust in other people particularly among never married/single and divorced, but also for poor psychological health among widowers/widows, compared to the majority category married/cohabitating men and women.

Social capital literature has suggested that increased divorce rates may partly explain the decreasing prevalence of high generalized trust in other people in the USA (Fukuyama, 1999, Putnam, 2000). This study will investigate generalized trust, i.e. trust in other people including people with whom the respondent only meet once and is likely never going to meet again, as a covariate in the relationship between marital status and all-cause, CVD, cancer, and other cause mortality.

In sum, most previous studies have not separated cohabitating from those living single. Furthermore, there is a comparative lack of studies regarding the association between marital status and cancer. Still, links between marital status and cancer may be hypothesized because unmarried individuals have been shown to have higher levels of cortisol and a flatter diurnal cortisol slope (Chin et al., 2017) which is linked to shorter survival among cancer patients (Sephton et al., 2013). Also, a spouse may improve the likelihood of contact with relevant health services (Björnelv et al., 2020). The hypotheses of this study are that statistically significant associations with higher all-cause, CVD, cancer, and other cause mortality among never married/single, divorced, and widowed men and women is higher than among married/cohabitating men and women, respectively.

The aim is to analyze associations between marital status and all-cause, CVD, cancer, and other causes mortality in multiple analyses, adjusting for confounders and other covariates.

## 2 Material and methods

### 2.1 Study population

In the autumn of 2008, a public health postal survey was conducted in Scania by Region Skåne, the regional authority responsible for the healthcare system and public health in southern Sweden, based on a stratified sample from the register population of adults 18–80 years, generated by *Statistics Sweden* from the national population register. This survey constitutes the baseline of the present study. There were three postal reminders. The questionnaire could also be completed online. The questionnaire was answered by 28,198 participants (54.1 % participation rate). A population weight was constructed by Statistics Sweden to compensate for differing response rates according to age, sex, and education. The baseline survey cross-sectional data was linked to prospective register mortality data by *the National Board of Health and Welfare* (*Socialstyrelsen*).

Ethical consent was approved by the Ethical Committee (*Etikprövningsnämnden*) in Lund (No. 2010/343).

### 2.2 Dependent variables

**Fig. 2:**
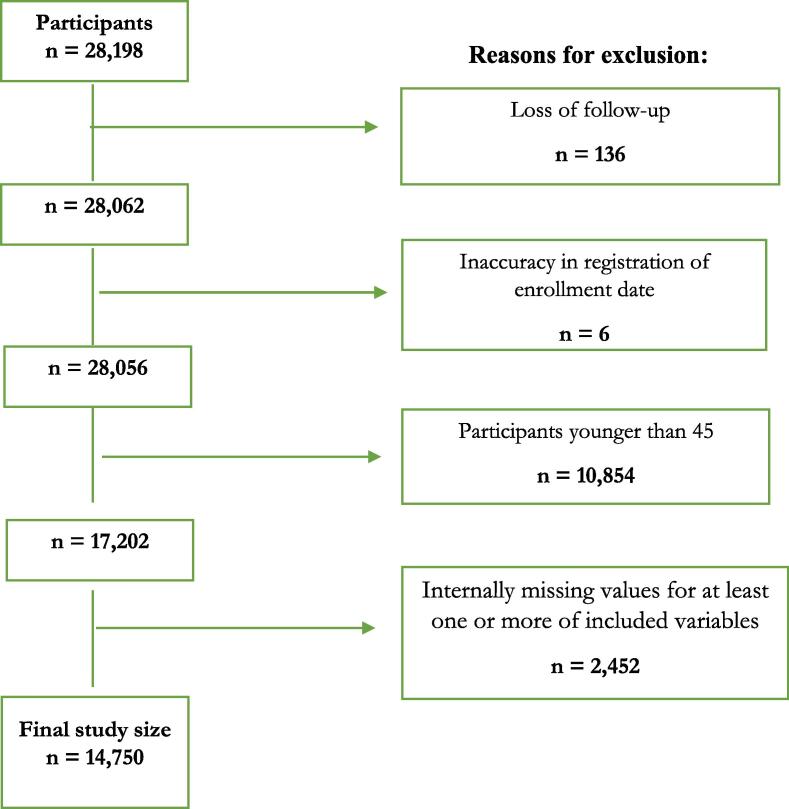
Flow chart of included study sample. The 2008 Public Health Survey of Scania, Sweden, with 2008–2016 follow-up.

Follow-up of mortality began on 27 August-14 November 2008 (the exact date defined by registration date of respondents’ answers) until 31 December 2016 (8.3 years follow-up), or until death. The present study included 14,750 participants, 6977 men and 7773 women aged 45–80 years. Only 14,750 participants aged 45–80 years were included in this study because the baseline is the sole time point for assessment of marital status, and only 51 deaths occurred in the age interval 18–44 years, which is the age interval where most changes in marital status occur over an 8.3-year follow-up. The present study included 14,750 participants after exclusion of 2452 respondents with internally missing values on any of the items from the baseline survey included in this study. Loss to follow-up included 136 persons in the entire age range 18–80, see Fig. 2. To classify deaths by diagnosis, the International Classification of Diseases 10 (ICD10) was used. The Swedish ten-digit person number system facilitates linking baseline survey data to prospective follow-up data. A third party (private company) conducted the linkage. The person numbers were deleted before delivery of the data to the researchers.

All-cause mortality and the three broad categories cardiovascular (I00-I98), cancer (C00-C97), and all other cause (other causes than I00-I98 and C00-C97) mortality were analyzed.

### 2.3 Independent variables

*Marital status* was categorized as married/cohabitating, never married/single, divorced, and widow/widower; *sex* was analyzed both collapsed and stratified by men and women in Table 3; *age* is a continuous variable; *country of birth* was defined as born in Sweden or other country; *chronic disease* was defined as “long-term disease, ailment, injury, disability or other weakness”. *Socioeconomic status (SES) by occupation* was defined into categories non-manual employees in higher, medium, and lower positions, skilled and unskilled manual workers, and self-employed, unemployed (job seekers), students, early retired (below 65), long-term sick leave, pensioners aged 65+, and unclassified. *Body mass index (BMI)* is a continuous variable; *smoking* included the alternatives daily, non-daily and non-smoker, dichotomized into daily smoking versus non-daily smoking/non-smoking; *leisure-time physical activity (LTPA)* is defined as regular exercise, moderate regular exercise, moderate exercise and sedentary lifestyle (less than two hours walking, cycling or equivalent per week) collapsing the first three alternatives (See Lindström et al., 2023). *Alcohol consumption* concerned frequency of consumption during the past twelve months including never, once a month or more seldom, 2–4 times a month, 2–3 times a week and at least 4 times a week. *Generalized trust in other people* was based on the item “Most people can be trusted” with the options “Do not agree at all”, “Do not agree”, “Agree” and “Agree completely”, collapsing the first two alternatives into “low trust” and the two latter “high trust”.

### 2.4 Statistics

Prevalence (%) was analyzed stratified by the four marital status categories for all variables except age and BMI that were analyzed as prevalence means. Differences for each variable according to the four marital status categories were analyzed with ANOVA-tests for continuous variables and chi-square tests for categorical variables (p-values) (Table 1).

**Table 1:** Characteristics of study population by marital status. The 2008 Public Health Survey in Scania, Sweden. Total population **n = 14 750 aged 45**–**80 years (6977 men and 7773 women)**. **Weighted prevalence.**

		**Marital status**				
		**Married/cohabitating**	**Never married/ single**	**Divorced**	**Widowed**	**p-value**
		n = 11191	n = 1136	n = 1606	n = 817	
		72.8 %	9.1 %	12.2 %	5.9 %	
						
**Death (n)**	773	103	159	162	<0.001	
**Age**, yrs: mean ± SD ^a^	59.5 ± 8.4 (59.3–59.8)	57.2 ± 8.7 (56.6–57.8)	59.9 ± 8.7 (59.3–60.5)	70.6 ± 7.7 (69.9–71.3)	<0.001	
**BMI:** mean ± SD ^a^	26.4 ± 3.8 (26.3–26.5)	26.4 ± 5.0 (26.0–26.7)	26.1 ± 4.6 (25.8–26.4)	26.5 ± 4.4 (26.1–26.9)	0.032	
**Men/Women^b^**						
	Men	55.3 (54.2–56.5)	58.5 (55.1–61.9)	42.3 (39.2–45.5)	26.5 (22.7–30.4)	<0.001
	Women	44.7 (43.5–45.8)	41.5 (38.1–44.9)	57.7 (54.5–60.8)	73.5 (69.6–77.3)	
**SES (Occupation)**^b^					<0.001	
	High non-manual	8.0 (7.5–8.6)	6.8 (5.2–8.4)	5.9 (4.4–7.4)	1.3 (0.5–2.1)	
	Medium non-manual	13.012.2–13.7)	11.1 (9.1–13.2)	10.3 (8.5–12.0)	3.2 (2.1–4.4)	
	Low non-manual	7.9 (7.3–8.5)	6.2 (4.6–7.8)	7.5 (5.9–9.0)	3.0 (1.5–4.5)	
	Skilled manual	10.0 (9.3–10.7)	8.8 (7.0–10.7)	9.5 (7.6–11.4)	3.2 (1.8–4.6)	
	Unskilled manual	10.6 (9.8–11.3)	13.1 (10.8–15.5)	10.9 (8.9–12.9)	2.6 (1.2–3.9)	
	Self-employed/farmer	7.9 (7.2–8.5)	7.0 (5.2–8.9)	5.2 (3.9–6.5)	1.3 (0.4–2.3)	
	Early retired	5.1 (4.6–5.7)	12.5 (9.9–15.1)	11.4 (9.4–13.4)	4.4 (2.7–6.0)	
	Unemployed	2.2 (1.8–2.5)	6.5 (4.4–8.6)	3.5 (2.2–4.9)	0.9 (0.2–1.6)	
	Student	0.3 (0.2–0.4)	1.2 (03–2.2)	0.8 (0.1–1.5)	------	
	Old age pensioner	31.9 (30.9–32.9)	23.1 (20.2–25.9)	31.3 (28.5–34.1)	78.4 (75.0–81.7)	
	Unclassified	2.0 (1.6–2.3)	2.4 (1.4–3.4)	1.6 (0.8–2.5)	0.5 (0.0–1.0)	
	Long-term sickleave	1.2 (0.9–1.5)	1.2 (0.5–1.9)	2.0 (1.1–3.0)	1.1 (0.0–2.2)	
**Born outside Sweden^b^**	16.5 (15.5–17.5)	14.6 (11.7–17.6)	24.0 (20.9–27.1)	16.7 (13.1–20.3)	<0.001	
**Chronic disease^b^**	32.9 (31.8–34.0)	40.8 (37.3–44.2)	46.1 (43.0–79.1)	40.5 (36.3–44.7)	<0.001	
**Low LTPA^b^**	12.8 (12.0–13.6)	19.5 (16.5–22.5)	16.8 (14.4–19.3)	18.6 (15.2–22.0)	<0.001	
**Daily smoking^b^**	13.9 (13.1–14.7)	22.7 (19.7–25.7)	24.9 (22.2–27.7)	14.2 (11.2–17.2)	<0.001	
**Alcohol drinking past year^b^**					<0.001	
Never	10.2 (9.5–10.9)	16.7 (13.9–19.4)	14.6 (12.3–16.8)	21.3 (17.7–25.0)		
Once a month or more seldom	17.9 (17.0–18.8)	27.5 (24.2–30.7)	27.6 (24.9–30.4)	25.4 (21.8–29.0)		
2–4 times a month	31.1 (30.1–32.1)	28.6 (25.3–31.8)	26.9 (24.1–29.7)	26.5 (22.9–30.1)		
2–3 times a week	29.2 (28.1–30.2)	17.5 (14.9–20.2)	21.3 (18.7–24.0)	14.6 (11.7–17.5)		
At least 4 times a week	11.7 (11.0–12.4)	9.7 (7.6–11.9)	9.6 (7.8–11.4)	12.2 (9.3–15.0)		
**Low generalized trust in other people^b,c^**						<0.001
	32.6 (31.5–33.7)	42.3 (38.6–45.9)	42.5 (39.4–45.7)	32.2 (28.2–36.1)		

Abbreviations: BMI, Body Mass Index; n, number of individuals; yrs, years; SD, Standard Deviation.

^a^ p-value: Independent samples ANOVA-test, 2-tailed.

^b^ p-value: Pearson Chi Square test, 2-sided.

^c^ Trust: The item “Most people can be trusted” included the alternatives do not agree at all, do not agree, agree and agree completely, and was dichotomized with the two first alternatives defined as low trust and the two latter as high trust.

The values in parentheses are 95% confidence intervals for mean or percent based on bootstrap method with 1000 number of replicates.

Crude (unadjusted) hazard rate ratios (HRRs) and 95 % confidence intervals (95 % CIs) were calculated for each independent variable with regard to all-cause mortality (Table 2).

**Table 2:** Hazard rate ratios (HRRs) with 95 % confidence intervals (95 % CIs) of all independent variables and all-cause mortality in bivariate unadjusted analyses. The 2008 Scania public health survey with 8.3 years follow-up 2008–2016. Men and women combined. Total population n = 14750 aged 45–80 years (6977 men and 7773 women). Weighted.

			**Crude**		
**Cause of death**		**Ref.**	**HRR**	**(95 % CI)**	**Number of deaths**
**All causes**					1197
**Marital Status**		Married/ cohabitating			
Never married/single			1.3*	(1.0–1.7)	
Divorced			1.7***	(1.3–2.1)	
Widowed			3.5***	(2.9–4.3)	
Sex		Men	0.6***	(0.5–0.7)	
Age			1.1***	(1.1–1.1)	
BMI			1.0**	(1.0–1.0)	
**Occupation (socioeconomic status)**		High non-manual			
	Medium non-manual		0.9	(0.5–1.8)	
	Low non-manual		1.1	(0.5–2.4)	
	Skilled manual		0.9	(0.4–2.0)	
	Unskilled manual		0.8	(0.4–1.6)	
	Self-employed/farmer		0.7	(0.3–1.6)	
	Early retired		5.7***	(3.1–10.5)	
	Unemployed		2.6*	(1.0–6.4)	
	Student		0.0**	(0.0–0.1)	
	Old age pensioner		8.3**	(4.7–14.5)	
	Unclassified		1.5	(0.4–5.8)	
	Long-term sick leave		6.6***	(3.0–14.5)	
Country of birth		Sweden	0.9	(0.8–1.2)	
Chronic disease		No	2.3***	(2.0–2.7)	
Leisure-time physical activity		Active	2.8***	(2.4–3.3)	
Daily smoking		No	1.4***	(1.2–1.7)	
**Alcohol drinking past year**		Never			
	Once a month or more seldom		0.9	(0.7–1.1)	
	2–4 times a month		0.4***	(0.3–0.5)	
	2–3 times a week		0.5***	(0.4–0.6)	
	At least 4 times a week		0.8	(0.7–1.0)	
**Generalized trust in other people**		Yes	1.2**	(1.1–1.4)	

Abbreviations: BMI, Body Mass Index.

Significance levels: * p < 0.05, ** p < 0.01, *** p < 0.001. Weighted Hazard Ratios. Bootstrap method (1000 replicates) for variation estimation.

Hazard rate ratios (HRRs) and 95 % confidence intervals (95 % CI:s) of all-cause, CVD, cancer and other causes mortality were analyzed with the married/cohabitating category as reference group. Five models 0–4 were created: model 0 was unadjusted, model 1 adjusted for age and sex, model 2 additionally adjusted for country of birth, SES by occupation and chronic disease, model 3 additionally adjusted for BMI, LTPA, smoking and alcohol consumption during the past year, and model 4 additionally adjusted for generalized trust in other people. The same models were analyzed stratified by sex. In these models, model 1 was adjusted for age (not sex), model 2 additionally for country of birth, SES by occupation and chronic disease, model 3 additionally for BMI, LTPA, smoking and alcohol consumption during the past year, and model 4 additionally adjusted for generalized trust in other people (Table 3). From the baseline in the autumn of 2008 follow-up days were counted either to death or last date of follow-up (31 December 2016). Sampling variability may be analysed with bootstrap analysis without distributional assumptions regarding the study population (SAS/STAT Survey Analysis, 2021). Bootstrap methods with 1000 numbers of replicates were used to obtain confidence intervals and p-values in order to ensure accurate variance estimation on weighted data. Proportionality tests for marital status and mortality were analyzed. An interaction term with time and marital status was included to test proportional hazards assumptions. Schoenfeld residuals were calculated for marital status and mortality. The Schoenfeld residuals compare the married/cohabitating category with the other three marital status categories collapsed (See Figure S1 in Supplement). The SAS software version 9.4 was utilized in the analyses.

**Table 3:** Hazard rate ratios (HRRs) with 95 % confidence intervals (95 % CIs) of all-cause, CVD, cancer and other cause mortality according to marital status in multiple adjusted models 0–4. The 2008 Scania public health survey with 8.3 years follow-up 2008–2016.

	**Model 0**		**Model 1**		**Model 2**		**Model 3**		**Model 4**		
**Cause of death**	**HRR**	**(95 %CI)**	**HRR**	**(95 %CI)**	**HRR**	**(95 %CI)**	**HRR**	**(95 %CI)**	**HRR**	**(95 % CI)**	**Number of** **deaths**
**Men and women combined. Total population n = 14750 aged 45**–**80 years (6977 men and 7773 women)**. **Weighted.**											
**All causes**											1197
Married/cohabitating	1.0		1.0		1.0		1.0		1.0		
Never married/single	1.3*	(1.0–1.7)	1.8***	(1.4–2.2)	1.6***	(1.3–2.0)	1.4**	(1.1–1.8)	1.4**	(1.1–1.8)	
Divorced	1.7***	(1.3–2.1)	1.9***	(1.5–2.4)	1.7***	(1.3–2.1)	1.4**	(1.2–1.8)	1.4**	(1.1–1.9)	
Widowed	3.5***	(2.9–4.3)	1.7***	(1.3–2.1)	1.6***	(1.3–2.0)	1.4**	(1.2–1.8)	1.4**	(1.2–1.8)	
**Men (n = 6977) aged 45**–**80 years**. **Weighted.**											
**All causes**											708
Married/cohabitating	1.0		1.0		1.0		1.0		1.0		
Never married/single	1.4*	(1.1–2.0)	2.1***	(1.5–2.8)	1.9***	(1.4–2.6)	1.7**	(1.2–2.3)	1.7***	(1.2–2.3)	
Divorced	2.0***	(1.5–2.7)	2.3***	(1.7–3.0)	1.9***	(1.4–2.6)	1.7***	(1.3–2.3)	1.7***	(1.3–2.3)	
Widowed	4.5***	(3.3–6.3)	2.0***	(1.4–2.7)	2.0***	(1.4–2.7)	1.7***	(1.3–2.3)	1.7***	(1.3–2.3)	
**Cardiovascular disease**											249
Married/cohabitating	1.0		1.0		1.0		1.0		1.0		
Never married/single	2.0**	(1.2–3.3)	3.0***	(1.8–4.9)	2.7***	(1.7–4.5)	2.4**	(1.4–4.1)	2.4**	(1.4–4.1)	
Divorced	2.6***	(1.6–4.3)	3.1***	(1.9–4.9)	2.6***	(1.6–4.3)	2.3***	(1.4–3.8)	2.3***	(1.4–3.8)	
Widowed	5.4***	(3.1–9.6)	2.2**	(1.2–4.1)	2.3**	(1.3–4.0)	2.0*	(1.2–3.5)	2.0*	(1.2–3.5)	
**Cancer**											247
Married/cohabitating	1.0		1.0		1.0		1.0		1.0		
Never married/single	0.7	(0.4–1.3)	1.0	(0.6–1.8)	1.0	(0.6–1.7)	0.9	(0.5–1.6)	0.9	(0.5–1.6)	
Divorced	0.6	(0.3–1.3)	0.7	(0.3–1.5)	0.6	(0.3–1.3)	0.5	(0.2–1.2)	0.5	(0.2–1.1)	
Widowed	2.2*	(1.1–4.4)	0.9	(0.5–1.9)	0.9	(0.4–2.0)	0.8	(0.4–1.8)	0.8	(0.4–1.8)	
**Other causes**											212
Married/cohabitating	1.0		1.0		1.0		1.0		1.0		
Never married/single	1.9*	(1.1–3.3)	2.7***	(1.6–4.6)	2.3**	(1.3–4.0)	2.0**	(1.1–3.5)	2.0*	(1.1–3.5)	
Divorced	3.4***	(2.2–5.4)	3.9***	(2.5–6.1)	3.2***	(2.1–5.0)	2.9***	(1.8–4.6)	2.9***	(1.8–4.6)	
Widowed	7.3***	(4.2–12.7)	3.3***	(1.9–5.9)	3.3***	(1.9–5.8)	2.7***	(1.6–4.8)	2.7***	(1.6–4.8)	
											
**Women (n = 7773) aged 45**–**80 years**. **Weighted.**											
**All causes**											489
Married/cohabitating	1.0		1.0		1.0		1.0		1.0		
Never married/single	1.0	(0.6–1.7)	1.1	(0.7–1.9)	1.0	(0.6–1.7)	0.9	(0.5–1.6)	0.9	(0.5–1.6)	
Divorced	1.6**	(1.2–2.1)	1.4*	(1.0–1.9)	1.3	(0.9–1.7)	1.1	(0.8–1.6)	1.1	(0.8–1.6)	
Widowed	4.0***	(3.0–5.2)	1.4*	(1.0–1.9)	1.3	(1.0–1.8)	1.2	(0.9–1.7)	1.2	(0.9–1.7)	
**Cardiovascular disease**											117
Married/cohabitating	1.0		1.0		1.0		1.0		1.0		
Never married/single	0.3	(0.0–11.1)	0.4	(0.0–13.3)	0.4	(0.0–14.7)	0.3	(0.0–13.3)	0.3	(0.0–13.1)	
Divorced	1.6	(0.9–3.0)	1.4	(0.7–2.6)	1.3	(0.6–2.4)	1.1	(0.5–2.1)	1.1	(0.5–2.1)	
Widowed	4.1***	(2.4–7.1)	1.3	(0.7–2.2)	1.2	(0.7–2.1)	1.0	(0.6–1.9)	1.0	(0.6–1.9)	
**Cancer**											221
Married/cohabitating	1.0		1.0		1.0		1.0		1.0		
Never married/single	0.8	(0.3–2.1)	0.9	(0.4–2.4)	0.9	(0.4–2.2)	0.8	(0.3–2.0)	0.8	(0.3–2.0)	
Divorced	**1.4**	(0.9–2.2)	1.2	(0.8–2.0)	1.2	(0.7–1.8)	1.0	(0.7–1.7)	1.0	(0.7–1.7)	
Widowed	3.3***	(2.1–5.1)	1.4	(0.9–2.3)	1.4	(0.9–2.2)	1.3	(0.8–2.1)	1.3	(0.8–2.1)	
**Other causes**											151
Married/cohabitating	1.0		1.0		1.0		1.0		1.0		
Never married/single	1.8	(0.8–4.1)	2.1	(1.0–4.8)	1.9	(0.8–4.1)	1.6	(0.7–3.7)	1.6	(0.7–3.7)	
Divorced	1.9*	(1.1–3.2)	1.6	(0.9–2.8)	1.5	(0.8–2.5)	1.3	(0.7–2.3)	1.3	(0.7–2.3)	
Widowed	5.1***	(3.1–8.3)	1.4	(0.8–2.5)	1.4	(0.8–2.4)	1.3	(0.7–2.3)	1.3	(0.7–2.3)	

Model 0 unadjusted.

Model 1 adjusted for sex and age when men and women were combined, adjusted for age (not sex) when the analyses were stratified by sex.

Model 2 additionally adjusted for socioeconomic status by occupation, country of birth and chronic disease.

Model 3 additionally adjusted for BMI, leisure-time physical activity, daily smoking and alcohol consumption.

Model 4 additionally adjusted for generalized trust in other people.

Significance levels: * p < 0.05, ** p < 0.01, *** p < 0.001

Weighted Hazard Ratios. Bootstrap method (1000 replicates) for variation estimation.

## 3 Results

Table 1 presents basic characteristics of study participants. A 72.8 % proportion of respondents were married/cohabitating, 9.1 % never married, 12.2 % divorced and 5.9 % widows/widowers. Never married was the significantly youngest category, followed by married/cohabitating, divorced and widows/widowers in consecutive and statistically significant order. Men were overrepresented in the marital status categories married/cohabitating and never married, while women were overrepresented in the categories divorced and widows/widowers. Differences in SES (according to occupation) by marital status were also observed. Respondents born abroad were significantly overrepresented in the divorced category. The never married/single, divorced, and widowed categories had significantly higher prevalence of chronic disease, low LTPA and daily smoking, than the married/cohabitating category. Never drinking during the past year was significantly more common in the widowed category than in the married/cohabitating and divorced categories. The never married/single and divorced had a significantly higher prevalence of low generalized trust than the married/cohabitating and widowed.

The unadjusted bivariate analyses in Table 2 show that the never married, divorced, and widowed had higher all-cause mortality than the married/cohabitating, and that women had lower all-cause mortality than men. All-cause mortality increased with age and higher BMI. Early retired, unemployed, old age pensioners and participants on long-term sick leave had higher all-cause mortality than non-manual employees in higher positions, while students had lower all-cause mortality. Participants who reported chronic disease, low LTPA, daily smoking and low generalized trust also displayed significantly higher all-cause mortality. Participants who consumed alcohol 2–4 times a month and 2–3 times a week had significantly lower all-cause mortality than the never consumption reference group.

Table 3 shows that never married/single, divorced and widowed men and women combined displayed significantly higher HRRs in models 0–4 compared to the married/cohabitating reference category. Among men, significantly higher HRRs of all-cause, CVD and other-cause mortality were observed throughout models 0–4 for the never married/single, divorced and widower categories compared to the married/cohabitating reference category. In contrast, no significant associations between never married/single, divorced and widowers and cancer mortality were observed among men in models 1–4. Divorced and widowed women displayed significant HRRs for all-cause mortality in model 1, but the HRRs for divorced and widowed women were not significant compared to married/cohabitating women in models 2–4. Never married/single women had no significant HRRs of all-cause mortality throughout models 0–4 compared to married/cohabitating women. Never married/single, divorced and widowed women displayed no significant HRRs of CVD, cancer and other cause mortality throughout models 1–4 compared to the married/cohabitating reference category.

Supplementary Figure S1 displays consistent and stable Schoenfeld residuals over time for marital status and all-cause mortality when married/cohabitating versus the other three marital status categories were analyzed. The interaction term between marital status and all-cause mortality over the 8.3 year period was p = 0.193, which indicates proportionality.

## 4 Discussion

Never married/single, divorced, and widowed men had significantly higher HRRs of all-cause, CVD and other cause mortality than the reference category married/cohabitating men throughout the multiple analyses. In contrast, no statistically significant associations were observed for cancer mortality among men. No significant associations were displayed for women in the multiple analyses. The results show that marriage/cohabitation is significantly associated with lower all-cause mortality among men but not among women. These results further support the notion that marriage/cohabitation has a stronger health protective effect for men than women (Wang et al., 2020, Robards et al., 2012, Manzoli et al., 2007). Potential explanations might include differences in biological, psychological, and social factors (Wang et al., 2020). Still, uncaring and unhelpful spousal behaviors may outweigh positive spousal behaviors and may contribute to poorer physical health (Bookwala, 2005).

SES and chronic disease patterns of marital status may indicate selection both regarding entry into and exit out of marriage/cohabitation. High LTPA and absence of smoking suggest protective effects. In contrast, the prevalence of low generalized trust in other people was significantly higher among never married/single and divorced than among married/cohabitating and widowed. Generalized trust in other people was significantly associated with all-cause mortality in the bivariate analyses (Table 2), but did not affect the hazard rate ratios in the final model of the multiple survival analyses.

The results also indicate that associations between marital status and cancer mortality were not statistically significant for neither men nor women throughout the multiple analyses. These results diverge from the statistically significant associations between marital status and all-cause, CVD and other causes mortality observed among men. Scarce previous studies indicating no associations between marital status and cancer mortality (Wang et al., 2020) are thus confirmed by this large, population-based prospective cohort study.

Some previous studies have reported problems separating single from cohabitating among the not married (Ben-Schlomo et al., 1993, Berntsen and Kravdal, 2012). In this study married and cohabitating were instead collapsed into a common category, which may be regarded as a strength considering the current practice of marriage and cohabitation in Sweden.

The finding of proportionality in the relationship between marital status and all-cause mortality over time suggests that marital status is a stable and consistent determinant of health. Tests of proportionality have been very scarce despite the recurrent use of Cox (survival) regression models.

The results further suggest that marital status should be regarded as a risk marker with lowest health risks for the married/cohabitating.

**Strengths and limitations**

This study is a prospective cohort study based on a large stratified random population sample. The participation rate (54.1 %) is comparable to other Swedish and western public health surveys, which have consistently indicated a decreasing trend in response rates over several decades. The respondent population has acceptable representativeness compared to the parameter population with some underrepresentation of the young, men, respondents born abroad and respondents with low education. Selection bias is not likely (Lindström and Rosvall, 2018). The present study did not assess the quality of marriage and there is thus a risk that some marriages, e.g. marriages characterized by conflict, have negative rather than positive effects on health (Bookwala, 2005). National level statistics from Sweden in 2008 indicate that in the age interval 45–80 years 57 % of men and women were married, 17 % not married, 18 % divorced and 7 % widowed (Statistics Sweden Gifta i Sverige, 2023). These proportions are not completely comparable to our study, because the 57 % proportion married nationally only contains those married, while the 72.8 % in our survey baseline contains both those married and those cohabitating without being married. Correspondingly, the 17 % proportion not married in the national data partly contains people not married but cohabitating, which is common in Sweden. The 18 % proportion of divorced nationally is higher than the 12.2 % proportion in this study, which may also partly be explained by cohabitation. Finally, the 7 % proportion of widowed nationally is close to the 5.9 % proportion in this study. It may still be possible that married/cohabitating is somewhat more common in Scania in southern Sweden than in Sweden nationally, while the proportion of divorced is somewhat lower. It is still highly likely that these differences do not alter the mechanisms behind the associations between marital status and mortality.

The present study included only participants aged 45–80 years. All independent variables including marital status were assessed only in the autumn of 2008. This means that marital status, which is a time dependent variable, was treated as a time-fixed variable over the 8.3-year follow-up period in our analyses. The age-interval 18–44 was excluded because only 51 deaths occurred in this age interval, which is also the age interval where most changes in marital status occur over an 8.3-year follow-up. All the other 1197 deaths over 8.3-year follow-up period occurred among participants in the age-interval 45–80 years at baseline.

Socioeconomic status (SES) includes the dimensions education, income and occupation. These dimensions are not identical but importantly correlated. The survey conducted in 2008 included no income item and the education item had a considerably higher number of internally missing than the SES item included in the analyses. The education item would substantially increase the number of internally missing if included in the analyses without affecting the general results and conclusions. The LTPA item is regarded as sufficiently valid compared to the golden standard measuring four-day whole-day calorimetry, heart rate (monitoring), and double-labelled water (Wareham et al., 2003). Items measuring smoking are generally regarded as valid and reliable (Wells et al., 1998). The generalized trust in other people item has previously been defined, validated and analyzed internationally (see e.g. Putnam, 1993, Putnam, 2000). Validity of causes of death has been investigated in several Swedish studies. The validity of Swedish cause of death data is high for many but not all diagnoses. Still, the proportion of autopsies has declined for decades resulting in a decrease in validity (Ludvigsson et al., 2011, Rosendahl et al., 2021). Validity of cancer diagnoses is still high, with correct classification at the level of specific diagnosis at 90 %, and so is validity of cardiovascular diseases, with correct classification at the level of specific diagnosis at 86 %. In contrast, validity of some other diagnoses such as e.g. chronic obstructive pulmonary disease (COPD) and other pulmonary diseases as underlying diagnoses is considerably lower with 47 % correct classification at the level of specific diagnosis (Brooke et al., 2017). As groups of aggregate causes of death diagnoses, correct classification into the aggregate CVD and aggregate cancer groups is probably higher than 90 % and 86 %, respectively, because misclassification of specific diagnoses may also occur within each aggregate group. The other cause of death group seems more problematic with regard to validity, but given the high validity in the two former groups of causes of death from CVD and cancer the lower validity among the causes of death within the aggregate group other diagnoses may be a smaller problem in this study.

Multiple survival (Cox) regression analyses with relevant cofounders and covariates including demographics, SES (by occupation), chronic disease, BMI, LTPA, smoking, alcohol consumption and generalized trust in other people were conducted.

## 5 Conclusion

Never married/single, divorced, and widowed men had significantly higher hazard rate ratios (HRRs) of all-cause mortality throughout the multiple survival analyses than married/cohabitating men. Analyses of cause-specific mortality showed significant results for CVD and other cause mortality but not statistically significant HRRs for cancer mortality throughout the multiple analyses. No significant associations were displayed for women in the multiple analyses. There are strong and significant associations between marital status and mortality among men but not among women. Associations between marital status and cancer mortality are not statistically significant with low effect measures throughout the multiple analyses among both men and women.

## Funding

This study was funded by Vetenskapsrådet (K2014-69X-22427-01-4) (data collection) and the Swedish ALF Government Grants (2023-2026/ Martin Lindström).

## CRediT authorship contribution statement

**Martin Lindström:** Conceptualization, Main writing, Main review and editing, Formal analysis, Main validation. **Mirnabi Pirnouzifard:** Data curation, Formal analysis, Methodology, Software, Writing – review & editing. **Maria Rosvall:** Validation, Writing – review & editing. **Maria Fridh:** Validation, Writing – review & editing.

## Declaration of Competing Interest

The authors declare that they have no known competing financial interests or personal relationships that could have appeared to influence the work reported in this paper.

## Data Availability

The authors do not have permission to share data.
