# Supplementary material for: Marital status and cause-specific mortality: A population-based prospective cohort study in southern Sweden
Source: Prev Med Rep. 2023 Dec 9;37:102542. doi: 10.1016/j.pmedr.2023.102542 (PMC10758969; doi:10.1016/j.pmedr.2023.102542)
Supplement: Supplementary data 1 [file mmc1.docx]

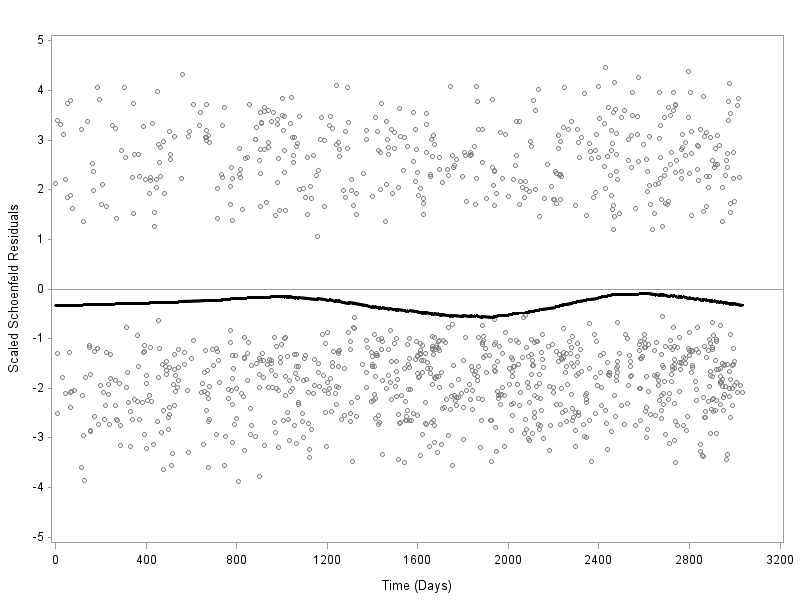


Figure S1. Schoenfeld residuals for men and women according to all-cause mortality and marital status (married/cohabitating against the three other marital status categories collapsed) over the 8.3-year period. The 2008 -2016 Scania public health survey with 8.3 years follow-up. Men and women combined (n=14750). The proportionality test with interaction term between marital status and all-cause mortality across the 8.3-year period is not significant, *p=0.193*, which indicates proportionality.
